# IMPACTS OF INTERGENERATIONAL CARE ON FUTURE CARE EXPECTATIONS: A SURVEY-EXPERIMENT STUDY OF AGING SANDWICH CARERS

**DOI:** 10.1093/geroni/igae098.4330

**Published:** 2024-12-31

**Authors:** Xue Bai, Shuai Zhou

**Affiliations:** The Hong Kong Polytechnic University, Hong Kong, Hong Kong, China (People’s Republic); The Hong Kong Polytechnic University, Hong Kong, Hong Kong, China (People’s Republic)

## Abstract

**Abstract background:**

An increasing number of people in their 50s and 60s are sandwiched between surviving parents and children. Numerous ageing adults often provide support for older and/or younger generations while also facing their own challenges and stereotypes associated with the transition to old age.

**Objectives:**

This study examined the effects of multi-dimensional, multi-directional care provision on future care expectation among sandwich carers.

**Design and methods:**

We implemented three factorial survey experiments within a large-scale representative survey of 1,500 sandwiched carers aged 50 and 69 (Mean age = 58.86±5.47) in Hong Kong. Care expectation was assessed in hypothetical scenarios across three key domains: financial, instrumental, and emotional care. Ordered logistic regression and multi-nominal logistic regression models were used.

**Results:**

Downward support to children was positively associated with filial care expectations, especially emotional support (OR = 1.45; 95% CI = [1.21, 1.73], p< 0.001). Sandwiched carers’ SES was negatively associated with expectations for financial (OR = 0.75; 95% CI = [0.62, 0.89], p< 0.01) and instrumental care (OR = 0.85; 95% CI = [0.71, 1.01], p=0.068) from children. Providing instrumental support to both parents and children reduced the expectation for future instrumental care. Having sons and greater socioeconomic resources increased the preference for self-care, while downward support reduced it.

**Discussion:**

Downward support to children strengthens filial care expectations among sandwich carers, especially mothers. However, providing instrumental support to both parents and children, along with SES, promotes the pursuit for self-care. It is crucial to enhancing sandwich carers’ image, caregiving capabilities and later-life preparations.

